# Cotranslational recruitment of ribosomes in protocells recreates a translocon-independent mechanism of proteorhodopsin biogenesis

**DOI:** 10.1016/j.isci.2021.102429

**Published:** 2021-04-20

**Authors:** Ross Eaglesfield, Mary Ann Madsen, Suparna Sanyal, Julien Reboud, Anna Amtmann

**Affiliations:** 1 Institute of Molecular, Cell and Systems Biology, University of Glasgow College of Medical, Veterinary and Life Sciences, Glasgow G12 8QQ, UK; 2 Department of Cell and Molecular Biology, Uppsala University, S-751 24 Uppsala, Sweden; 3 Division of Biomedical Engineering, University of Glasgow School of Engineering, Glasgow G12 8QQ, UK

**Keywords:** Molecular Biology, Cell Biology, Synthetic Biology

## Abstract

The emergence of lipid membranes and embedded proteins was essential for the evolution of cells. Translocon complexes mediate cotranslational recruitment and membrane insertion of nascent proteins, but they already contain membrane-integral proteins. Therefore, a simpler mechanism must exist, enabling spontaneous membrane integration while preventing aggregation of unchaperoned protein in the aqueous phase. Here, we used giant unilamellar vesicles encapsulating minimal translation components to systematically interrogate the requirements for insertion of the model protein proteorhodopsin (PR) – a structurally ubiquitous membrane protein. We show that the N-terminal hydrophobic domain of PR is both necessary and sufficient for cotranslational recruitment of ribosomes to the membrane and subsequent membrane insertion of PR. Insertion of N-terminally truncated PR was restored by artificially attaching ribosomes to the membrane. Our findings offer a self-sufficient protein-inherent mechanism as a possible explanation for effective membrane protein biogenesis in a “pretranslocon” era, and they offer new opportunities for generating artificial cells.

## Highlights

• Generated a simple artificial cell model for membrane protein insertion
• We identified protein-inherent control of translational targeting without chaperones
• Ribosomes, artificially tethered to GUVs increased membrane protein insertion

## Graphical abstract

**Figure:**
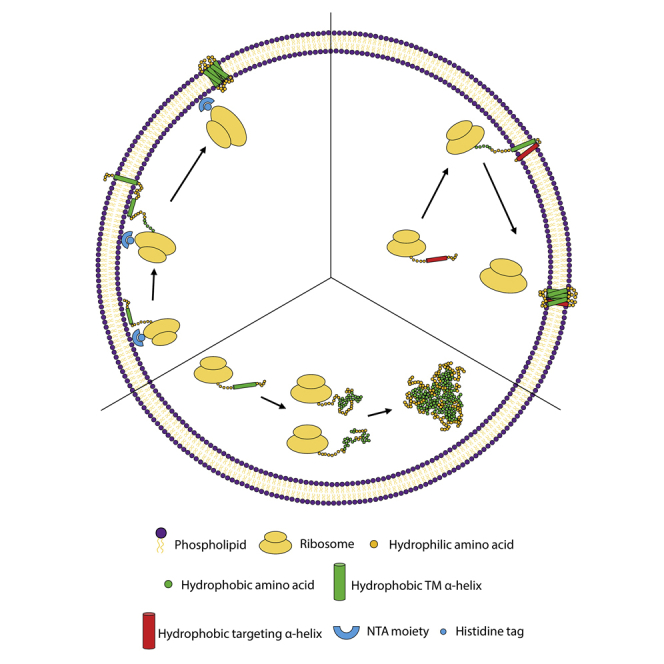

## Introduction

How cellular life first emerged on Earth remains one of the most fundamental questions in biology. The evolution of lipid-based membranes with embedded protein components is considered a crucial step in the emergence of primordial cells (Lane and Martin, 2012). Alpha-helical membrane proteins are ubiquitous and perform a startling number of essential tasks. Owing to their predominantly hydrophobic nature, these proteins readily integrate into the amphiphilic environment of a cell membrane (White and Wimley, 1999).

A four-step thermodynamic model for spontaneous insertion of α-helical membrane proteins has been proposed and rigorously tested (Cymer et al., 2015; MacKenzie, 2006; White and Wimley, 1999; Wimley and White, 1996). Briefly, an unstructured hydrophobic peptide chain experiences a large free-energy-barrier-preventing membrane insertion (Wimley and White, 1996). The adoption of hydrogen-bonded α-helical structure lowers this free-energy-cost-making membrane partitioning favorable (Almeida et al., 2012; BenTal et al., 1997; Ladokhin and White, 1999). The bilayer interface region, a complex chemical environment dominated by lipid headgroups, facilitates the adoption of α-helical structure and thus aids in the spontaneous integration process (Ladokhin and White, 1999; Ulmschneider et al., 2011). However, in the aqueous environment, away from the bilayer interface region, aggregation of hydrophobic peptides predominates and inevitably leads to permanently aggregated states unable to adopt native structure and partition into the bilayer (Cymer et al., 2015).

Modern cells have evolved various chaperoning pathways to prevent aggregation in the cytoplasm and facilitate membrane insertion (Steinberg et al., 2018). The signal recognition particle (SRP) chaperoning pathway is the most common and facilitates the cotranslational targeting and insertion of the vast majority of α-helical membrane proteins. Via interactions with its membrane bound receptor, SRP positions translating ribosomes in close proximity to a membrane-embedded insertion apparatus such as the Sec translocon (Akopian et al., 2013; Rapoport, 2007). Given the complexity of this machinery, it is likely that membrane proteins were originally inserted into the membrane spontaneously and that mechanisms such as SRP targeting and the Sec translocon evolved later, becoming essential as compartmentalization and functional diversity of cells increased (Bohnsack and Schleiff, 2010; Pohlschroder et al., 2005).

Given the propensity for detrimental aggregation in aqueous environments, the vast majority of α-helical membrane proteins are inserted into the membrane cotranslationally (Cymer and von Heijne, 2013; Luirink et al., 2012). The efficient recruitment of translating ribosomes to the membrane becomes crucial as cell size and complexity increase (Soga et al., 2014). This raises the question whether α-helical membrane proteins that emerged before the evolution of chaperoning/translocon systems have inherent features that facilitate membrane recruitment and insertion. Previous work has demonstrated that many membrane proteins can spontaneously integrate and fold into simple lipid membranes (Findlay et al., 2016; Harris et al., 2017; Pellowe and Booth, 2019; Soga et al., 2014). However, whether protein-inherent features of the emerging nascent chain affect recruitment of ribosomes to the membrane and thus tip the balance from aggregation toward integration remains to be investigated. Such a mechanism would represent an intriguing theory for how early cells could have overcome the lack of a dedicated chaperoning and translocon system. A bottom-up approach using artificial, cell-mimicking systems offers excellent opportunities to probe these hypotheses surrounding membrane protein biogenesis in the absence of a translocon (Xu et al., 2016).

To examine the possibility of protein-inherent membrane recruitment, we turned to the recombinant “cell-free” protein synthesis system known as the Protein synthesis Using Recombinant Elements (PURE). This system allows the investigation of protein folding in a minimal environment devoid of any chaperoning proteins or other insertion mediating factors (Kuruma and Ueda, 2015; Shimizu et al., 2001). When coupled with liposomes and lipid nanodiscs, this system has proven valuable for enhancing our understanding of the spontaneous membrane protein insertion process (Berhanu et al., 2019; Findlay et al., 2016; Harris et al., 2017; Matsubayashi et al., 2014). The PURE system can also be readily encapsulated within cell-size mimicking giant unilamellar vesicles (GUVs) and has been used in this way to highlight the importance of the vesicle surface area/volume ratio for membrane protein insertion/aggregation (Soga et al., 2014). To date, the focus of such studies has been on the insertion process itself, while the question of how these highly hydrophobic proteins avoid aggregation in a cell-mimicking context has not been thoroughly addressed.

Here, we have developed methods to interrogate the cotranslational recruitment of ribosomes to the membrane in a minimal context. We encapsulated PURE reactions within GUVs and used confocal imaging to investigate the minimal requirements for membrane localization and insertion of the model α-helical membrane protein proteorhodopsin (PR) (Beja et al., 2000). We could show that the cotranslational recruitment of ribosomes to the membrane is the major determinant of the fate of proteorhodopsin in GUVs even in the absence of specific chaperoning and targeting pathways. We found that cotranslational ribosome recruitment is an inherent effect driven by the physical characteristics of the N-terminal hydrophobic domain of PR. The importance of this mechanism was further proven by artificially tethering the ribosomes to the membrane, which rescued the insertion of mislocalized PR lacking the N-terminal domain. The fundamental insights and techniques developed here can now be used to investigate bottom-up membrane protein assembly in a more biologically relevant context and will allow the expansion of the current tool set for the generation of simple artificial cell models.

## Results

### The N-terminal domain of *de novo* synthesized proteorhodopsin enhances membrane insertion in cell-size-mimicking giant vesicles

**Figure 1:**
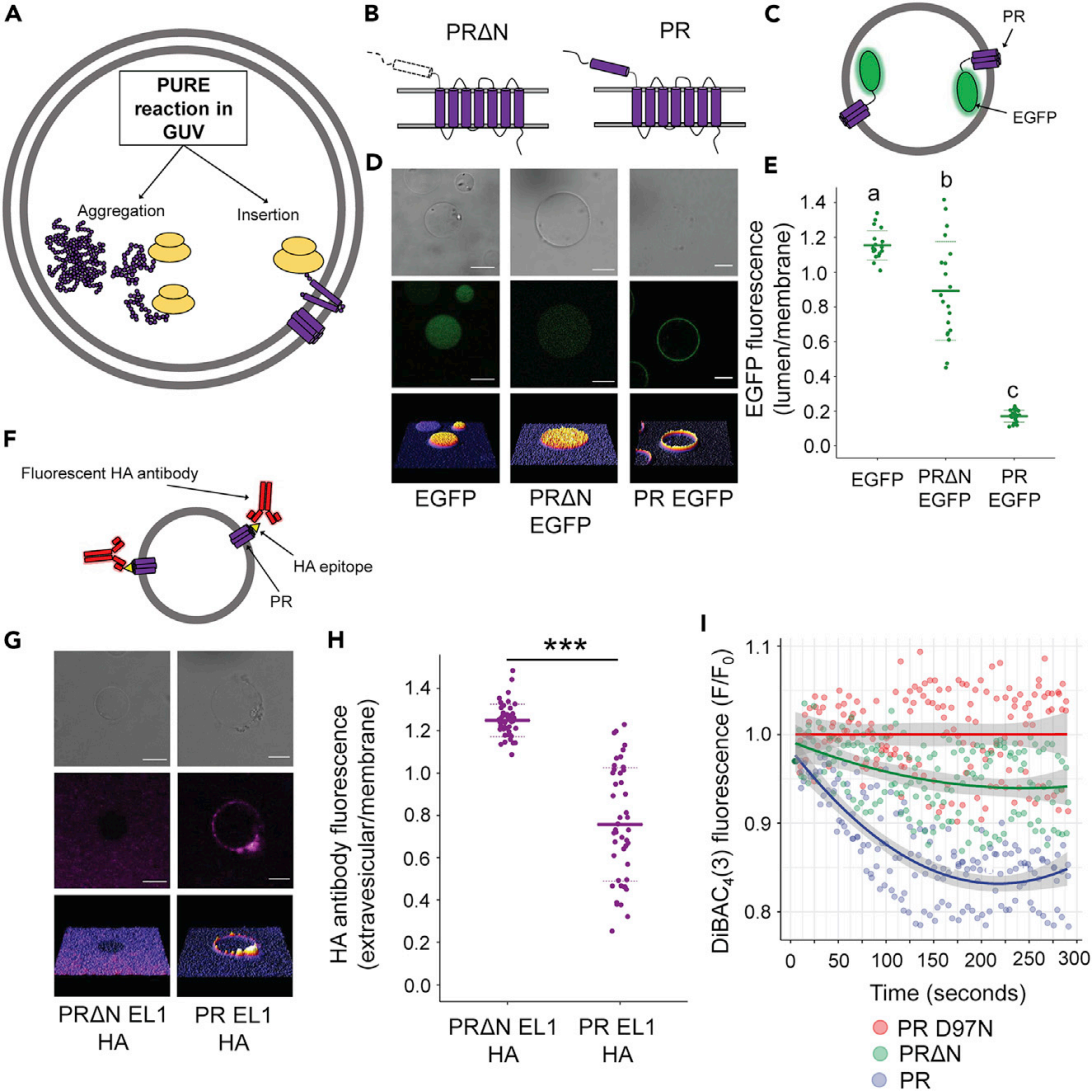
The N-terminal domain of proteorhodopsin (PR) is required for membrane localization and insertion (A) Schematic indicating the two potential fates (aggregation and insertion) of membrane proteins synthesized in cell-size-mimicking biomimetic systems. (B) Schematic representation of the topology PR with the N-terminal hydrophobic domain removed (PRΔN) and full-length PR. (C) Experimental system for analysis of EGFP localization. (D) Confocal microscopy images of GUVs after internal *de novo* protein synthesis of soluble EGFP (left), PRΔN-EGFP (center) and PR-EGFP (right). From top to bottom: brightfield image, fluorescence emission and 3-dimensional representations of fluorescence. Scale bars are 10 μm. (E) Lumen/membrane fluorescence intensity ratio derived from radial profiles of EGFP fluorescence emission of individual GUVs. Mean values (solid lines) and standard deviations (dashed lines) are shown for 20 individual GUVs (filled circles). Different letters represent statistically significant differences (p < 0.001; one-way ANOVA using Tukey [HSD] post-hoc analysis). (F) Experimental system for analysis of florescent HA antibody binding. (G) Confocal images of GUVs after protein synthesis and incubation with Alexa-Fluor-647-conjugated HA antibody. Images are organized as in (D). Scale bars are 10 μm. (H) Extravesicular/membrane fluorescence intensity ratio derived from radial profiles of Alexa-Fluor-647-conjugated HA antibody fluorescence of individual GUVs. Mean values (solid lines) and standard deviations (dashed lines) are based on 45 individual GUVs (filled circles). Asterisks represent statistical significance (P < 0.001) using a two-sample t test assuming equal variance. Data were normalized against a control ratio taken from GUVs expressing PR-EGFP with no HA epitope. (I) Fluorescence intensity of DiBAC_4_(3) in the membrane of GUVs containing a non-functional PR mutant (D97N), PR without the N-terminal domain (PRΔN) and full-length PR. Individual vesicles were analyzed every 5 s under constant excitation with a 488-nm laser to excite both DiBAC_4_(3) and PR. 12 GUVs from 3 individually prepared experiments were analyzed for each protein construct with results showing the mean value from each experiment. Data are fitted with second order polynomials and 99% confidence intervals. All data were normalized to PRD97N data to account for photobleaching of DiBAC_4_(3). See also Figures S1–S6.

As a minimal, cell-size-mimicking chassis, we used GUVs generated by the droplet transfer approach (Altamura et al., 2017; Noireaux and Libchaber, 2004), encapsulating PURE system components within the vesicle lumen. Plasmid DNA constructs containing the truncated or full-length forms of PR (PRΔN and PR, respectively) (Figures 1A, 1B, and S1) were then supplemented into inner solutions allowing the investigation of protein localization, insertion, and function using a range of confocal fluorescence microscopy assays. Enhanced green fluorescent protein (EGFP) fusion tags were first added to the C-terminus (Figure 1C) and the ratio of fluorescence obtained from the lumen and membrane of individual vesicles (compared with a soluble EGFP control Figure S2) enabled us to assess protein localization.

PRΔN lacking the N-terminal domain exhibited weaker membrane localization when compared with full-length PR, which showed a remarkably high association to the membrane (Figures 1D and 1E). We analyzed the effects of GUV size on membrane localization by performing a correlation analysis. A moderate positive correlation was observed for both PR and PRΔN suggesting that increasing vesicle size slightly reduced PR localization. Heterogeneity between individual GUVs was to be expected given that the efficiency of the encapsulation process is not saturated (Figure S3). Importantly, the observed membrane localization was indicative of protein insertion into the membrane as determined through the use of an antibody-binding assay (Figure 1F). Externally supplied fluorescently labeled hemagglutinin (HA) antibody showed preferential membrane localization when an HA epitope was introduced into the first periplasmic/extravesicular loop of PR (Figure S4), when compared with PR containing an HA epitope insertion in the third intracellular loop and to all PRΔN constructs (Figures 1G, 1H, and S5).

A functional analysis was carried out to further correlate the observations made regarding localization and insertion. A fluorescent indicator of membrane potential, DiBAC_4_(3), was introduced into GUV membranes and dye emission was tracked upon the excitation of PR. The nonfunctional PR mutant D97N, which is incapable of proton transport (Pfleger et al., 2009), was used as a negative control. Full-length PR containing the N-terminal domain exhibited enhanced function in GUVs compared with PRΔN. However, PRΔN still showed low levels of functionality indicating that at least a small proportion of the protein was inserted and folded correctly in GUV membranes (Figures 1I and S6).

These combined results show that full-length PR is recruited and inserted into the membrane of GUVs in the absence of any soluble chaperones or translocon proteins, highlighting a crucial role for the N-terminal domain, which may form an eighth membrane-embedded α-helix.

### The N-terminal hydrophobic domain of PR acts as a membrane anchor and facilitates downstream helix insertion

**Figure 2:**
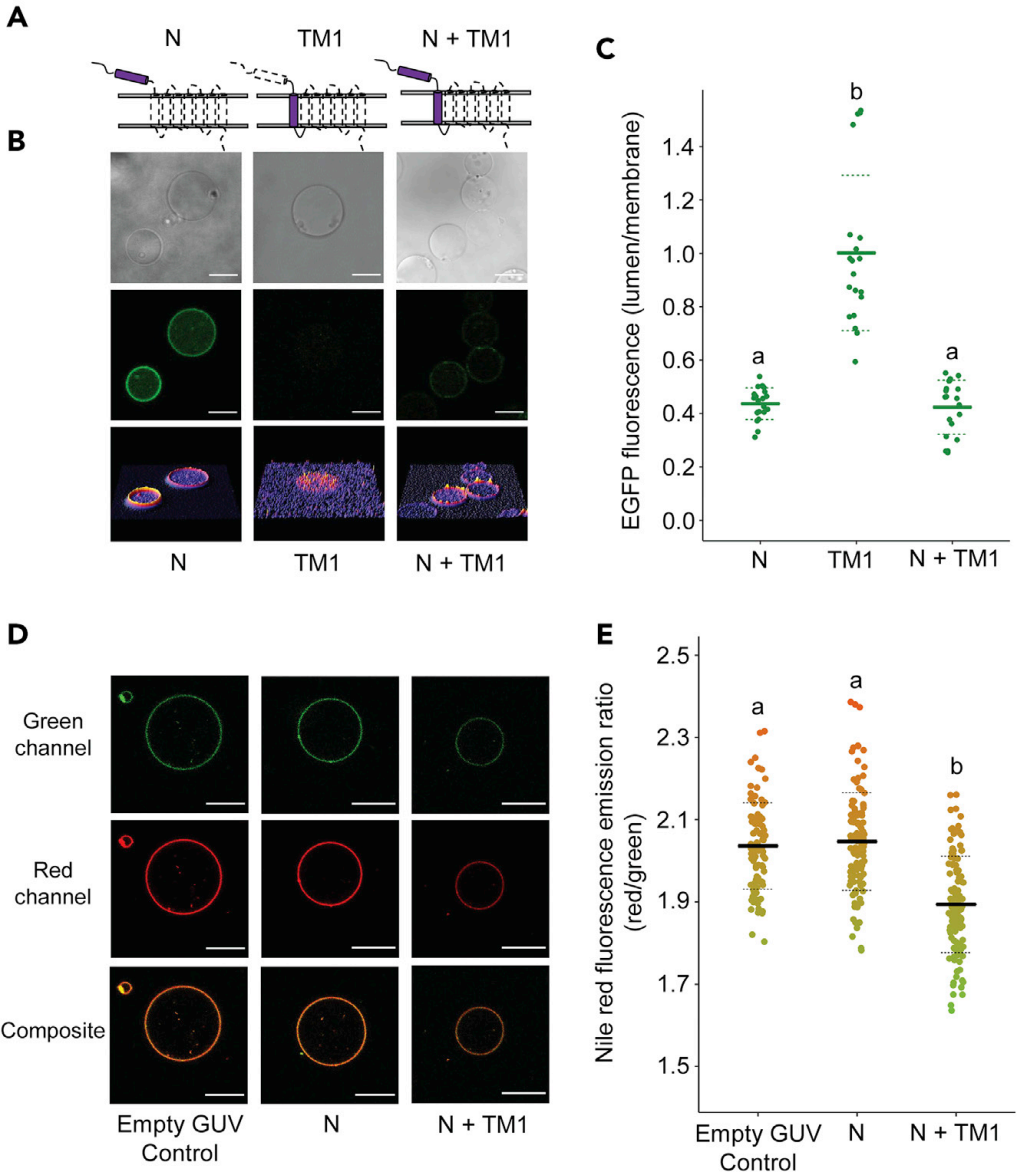
The N-terminal domain of PR facilitates downstream helix insertion (A) Schematic showing the regions of PR used in the experiments. (B) Confocal images of GUVs after protein synthesis. Scale bars are 10 μm. (C) Lumen/membrane fluorescence intensity ratio derived from radial profiles of EGFP fluorescence emission of individual GUVs. Mean values (solid lines) and standard deviations (dashed lines) are shown for 20 individual GUVs (filled circles). Different letters represent statistically significant differences (p < 0.001; one-way ANOVA using Tukey [HSD] post hoc analysis). (D) Confocal images of GUVs after protein synthesis and treatment with 0.1 μM of nile red. Images show fluorescence emission from 510–590 nm (green channel), 650–750 nm (red channel) and a composite of both emission channels. Scale bars are 10 μm. (E) Quantification of nile red fluorescence ratio (red/green) for GUVs. Mean values (solid lines) and standard deviations (dashed lines) are shown for ≥60 individual GUVs (filled circles). Different letters represent statistically significant differences (p < 0.001; one-way ANOVA using Tukey [HSD] post hoc analysis). See also Figure S7.

To further study the function of the N-terminal domain of PR, constructs were generated allowing PURE-mediated synthesis of the N-terminal domain in isolation (N), the first transmembrane helix (TM1), and the N-terminal domain together with the first transmembrane helix (N + TM1), all with a C-terminal EGFP fusion (Figure 2A). The N-terminal domain alone (N) was able to localize to the membrane despite the absence of downstream transmembrane helices, while TM1 exhibited very little membrane localization. The membrane localization of TM1 was recovered when the N-terminal domain was present (N + TM1) (Figures 2B and 2C).

The depth of peptide insertion into the hydrophobic core of the bilayer was then probed using the lipophilic environment-sensitive fluorescent probe nile red which reports on the fluidity of lipid tail groups (Mukherjee et al., 2007). Nile red undergoes a blue-shift in fluorescence emission in response to increasing membrane rigidity. We confirmed the sensitivity of nile red in GUVs with a range of POPC:cholesterol ratios (Figure S7) and then measured the red/green fluorescence emission ratio of nile red in the membranes of GUVs without (control) or with the PURE system enclosed. The N-terminal domain of PR alone (N) did not alter the lipid environment of the GUVs when synthesized in isolation, despite the high levels of membrane localization previously observed. By contrast, N + TM1 caused a significant decrease of red/green ratio indicating that it was able to constrain the lipid tails at the outer leaflet and increase membrane rigidity (Figures 2C and 2D). These data suggest that the N-terminal hydrophobic domain alone does not penetrate deeply into the core of the bilayer but can facilitate the insertion of TM1 which is otherwise not inserted into the membrane despite its high relative hydrophobicity.

### Localization and insertion of *de novo* synthesized PR in GUVs requires cotranslational targeting

**Figure 3:**
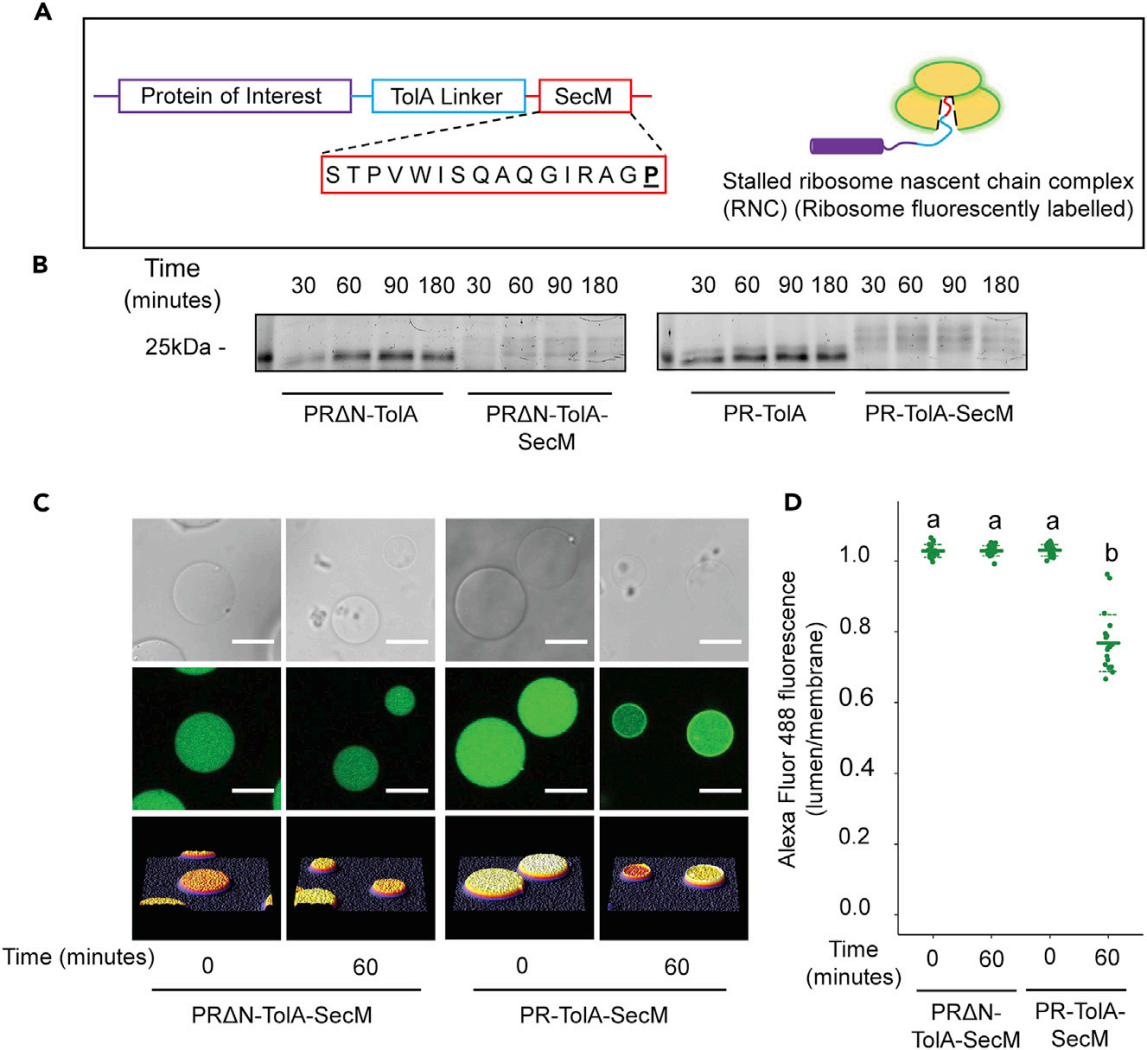
The N-terminal domain of PR recruits translating ribosomes to the membrane (A) Schematic representation of the construct used for stalling analysis and the experimental approach. The protein of interest is linked to an unstructured C-terminal linker sequence from the *E. coli* TolA protein and a stalling sequence from the *E. coli* SecM protein. The amino acid sequence of SecM is shown with the terminal proline responsible for translational stalling in bold and underlined. Fluorescently labeled, stalled RNCs can be visually tracked to probe for membrane enrichment. (B) SDS-PAGE time-series analysis of *in vitro* PURE reactions synthesizing PRΔN and PR with and without the SecM stalling sequence. Images are of the same gel stained with Coomassie or probed for BODIPY fluorescence. (C) Confocal images of GUVs encapsulating PURE reactions for PRΔN or PR supplemented with 1 μM of Alexa-Fluor-488-labeled ribosomes before and after a 60-min incubation at 37°C. Scale bars are 10 μm. (D) Lumen/membrane fluorescence intensity ratio derived from radial profiles of Alexa Fluor 488 fluorescence emission of individual GUVs. Mean values (solid lines) and standard deviations (dashed lines) are shown for 20 individual GUVs (filled circles). Different letters represent statistically significant differences (p < 0.001; one-way ANOVA using Tukey [HSD] post hoc analysis). See also Figures S8 and S9.

Our results indicate that the mechanisms involved are cotranslational and require ribosome recruitment to the membrane which in turn relies on protein-inherent features of the N-terminal hydrophobic domain. PR synthesized in the absence of a bilayer was unable to localize to posttranslationally supplied GUV membranes (Figure S8). Using a stalling peptide (SecM from *E. coli*), which causes translational arrest at a terminal proline residue, we generated constructs that result in the accumulation of stalled ribosome nascent chain complexes (RNCs). Figure 3 shows that stalled RNCs (fluorescently labeled) were only recruited to GUV membranes when the N-terminal hydrophobic domain was present. This behavior is consistent with previous results, both *in vivo* and *in vitro*, showing that most α-helical membrane proteins are inserted into the membrane cotranslationally (Cymer and von Heijne, 2013; Harris et al., 2017; Rapoport, 2007). We also confirmed that the mechanism is not linked to the membrane localization of mRNA (Jagannathan et al., 2014; Nevo-Dinur et al., 2011), regardless of the presence or absence of the coding sequence for the N-terminal hydrophobic domain (Figure S9). Consequently, the N-terminal domain could be used to guide translating ribosomes to the membrane, thus increasing the efficiency of membrane insertion over aggregation in the lumen.

### The artificial tethering of ribosomes enables the localization and insertion of PR lacking the N-terminal domain

In association with lipids, PR is known to adopt its native structure both *in vivo* and *in vitro* regardless of the presence of the N-terminal hydrophobic domain (Gourdon et al., 2008; Soto-Rodriguez and Baneyx, 2019). However, cotranslational ribosome membrane recruitment could become critical as vesicle size increases. If this is the case, one would predict that artificial tethering of ribosomes to the membrane would restore insertion of a PR lacking the N-terminal domain.

**Figure 4:**
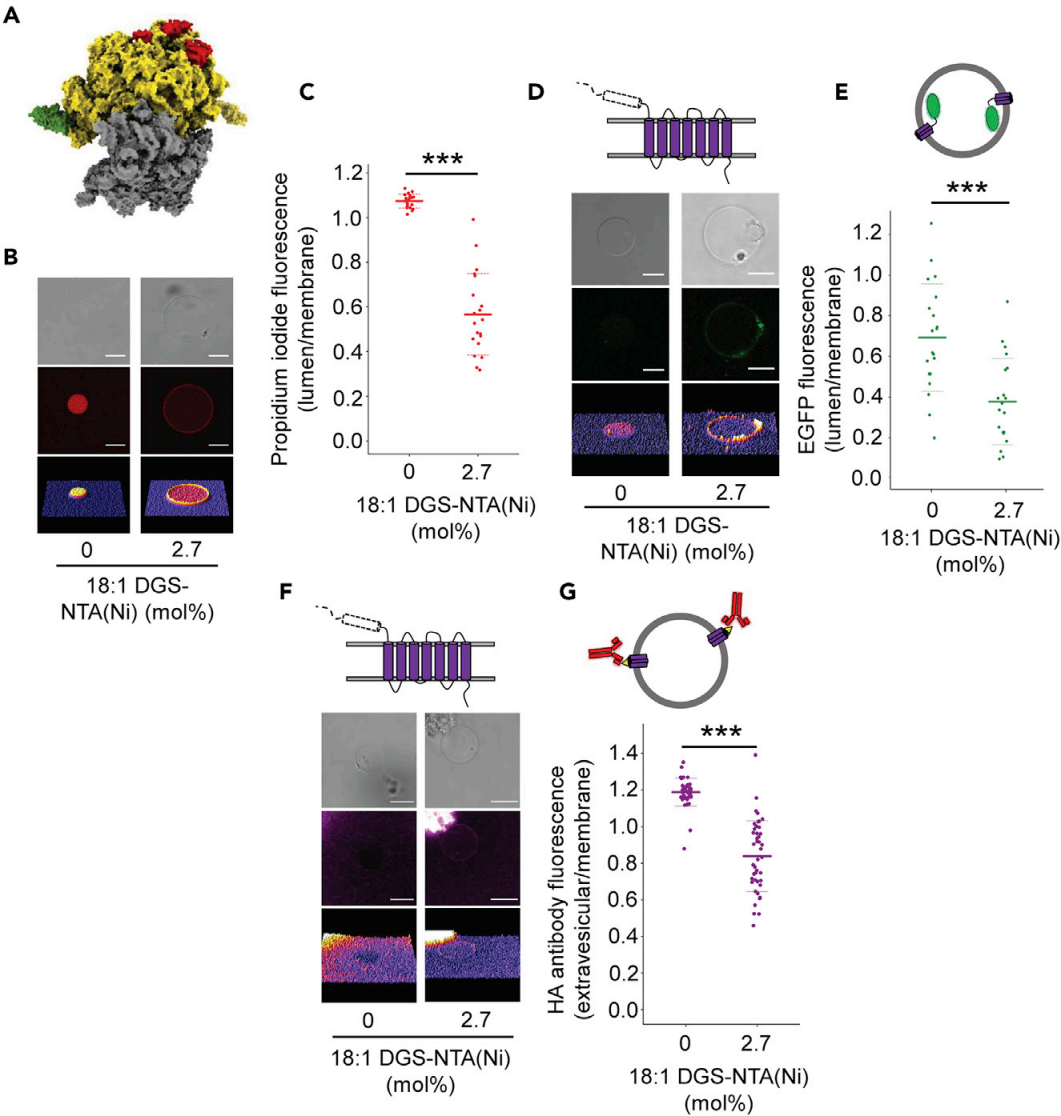
Pretranslational membrane tethering of ribosomes rescues the localization and insertion of PR lacking the N-terminal domain (A) Schematic representation of the His-tagged bacterial 70S ribosome complex (PDB entry 4V4P). Yellow and gray surfaces represent the 50S and 30S subunits, respectively; green and red surfaces represent the histidine-tagged L7/12 proteins and exit tunnel proteins, respectively. (B) Confocal images of GUVs encapsulating 1 μM of 70S ribosomes stained with 100 μg/mL propidium iodide (PI). GUVs were generated with and without 2.7 mol% DGS-NTA(Ni) in the membrane. Scale bars are 10 μm. DGS-NTA(Ni) tethers His-tagged ribosomes to the membrane. A control experiment was performed to ensure that the antibody did not bind directly to the NTA containing lipid (Figure S6). (C) Lumen/membrane ribosomal fluorescence intensity ratio derived from radial profiles of PI emission in individual GUVs. Mean values (solid lines) and standard deviations (dashed lines) are based on 20 individual GUVs (filled circles). Asterisks represent statistical significance (P < 0.001) using a two-sample t test assuming equal variance. (D) Confocal images of GUVs after synthesis of PRΔN-EGFP by His-tagged ribosomes tethered to the membrane (2.7) or not (0). Scale bars are 10 μm. (E) Lumen/membrane fluorescence intensity ratio derived from radial profiles of EGFP fluorescence emission of individual GUVs. Mean values (solid lines) and standard deviations (dashed lines) are shown for 20 individual GUVs (filled circles). Asterisks represent statistical significance (P < 0.001) using a two-sample t test assuming equal variance. (F) Confocal images of GUVs after synthesis of PRΔN-EL1HA by His-tagged ribosomes tethered to the membrane (2.7) or not (0) and incubation with Alexa-Fluor-647-conjugated HA antibody. Scale bars are 10 μm. (G) Extravesicular/membrane fluorescence intensity ratio derived from radial profiles of Alexa-Fluor-647-conjugated HA antibody fluorescence of individual GUVs. Mean values (solid lines) and standard deviations (dashed lines) are based on ≥ 45 individual GUVs (filled circles). Asterisks represent statistical significance (P < 0.001) using a two-sample t test assuming equal variance. Data were normalized against a control ratio taken from GUVs expressing PR-EGFP with no HA epitope. See also Figures S10 and S11.

Using vesicles doped with the synthetic lipid 1,2-dioleoyl-sn-glycero-3-[(N-(5-amino-1-carboxypentyl) iminodiacetic acid) succinyl] (nickel salt) (DGS-NTA(Ni)) (Peters et al., 2015), we attached modified 70S ribosome complexes from the *Escherichia coli* strain JE28 containing hexahistidine tags on the four L7/12 proteins of the large ribosomal subunit (Figure 4A) (Ederth et al., 2009), visualized using propidium iodide fluorescence staining of rRNA (Figures 4B, 4C, and S10). Functionality of the purified His-tagged ribosomes was confirmed by replacing commercially supplied ribosomes in bulk PURE reactions, resulting in the successful synthesis of α-hemolysin (Figure S11).

Enhanced membrane localization of PRΔN-EGFP was observed when ribosomes were tethered to the membrane (Figures 4D and 4E). Recovery of membrane insertion of PRΔSP was also confirmed by the HA epitope assay used previously (Figures 4F and 4G). A control experiment was performed to ensure that the antibody did not bind directly to the NTA-containing lipid (Figure S5). These experiments showed that the requirement of the N-terminal domain of PR for membrane localization and insertion can be replaced by artificially attaching the ribosomes to the membrane. The data indicate that the proximity of ribosomes to the membrane is a crucial limiting factor for the spontaneous, cotranslational insertion of PR into the membrane of cell-size-mimicking vesicles, at least when no receptor-mediated targeting mechanisms are present.

## Discussion

The use of bottom-up approaches to investigate the membrane protein biogenesis process have led to remarkable discoveries which begin to address some of the fundamental questions concerning the evolution of cellular membranes. It has long been argued that the final adopted structure of an α-helical membrane protein represents a thermodynamically favorable state (Cymer et al., 2015; Popot and Engelman, 1990). It is therefore logical to assume that many membrane proteins already contain within their amino acid sequences the necessary tools to overcome energetic barriers arising during membrane insertion. Thus, they may not require the assistance of sophisticated chaperoning and insertion complexes as long as the cellular context is simple, for example, lacking functionally diverse cellular compartments. Recent studies have indeed provided some evidence that translocons are not an absolute requirement for the insertion and folding of many membrane proteins, at least in simplified biomimetic systems, and are only needed for the insertion of proteins with large extracellular soluble domains (Baars et al., 2008; Berhanu et al., 2019; Matsubayashi et al., 2014). We have shown here that proteorhodopsin (PR), a model α-helical membrane protein is able to spontaneously integrate into a simple lipid membrane of giant vesicles in the absence of a translocon or chaperoning proteins.

If a purely thermodynamically driven pretranslocon mechanism of insertion prevailed early in evolution (Pohlschroder et al., 2005), membrane recruitment of translating ribosomes was likely crucial to avoid misfolding of aggregation-prone hydrophobic proteins before they came into contact with a membrane. Some modern cells still contain clues that hint at the importance of such a mechanism. For example, yeast mitochondrial ribosomes are permanently attached to the inner mitochondrial membrane which lacks a Sec translocon (Glick and VonHeijne, 1996; Jia et al., 2003). This attachment facilitates the insertion of membrane proteins encoded by the mitochondrial DNA (Szyrach et al., 2003).

Given the structural ubiquity of rhodopsins throughout all domains of life and thus the likely ancient nature of such proteins, PR seems to be a good model to investigate this hypothesis. We have shown here that the N-terminal hydrophobic domain of PR is critical for the localization and insertion of the membrane-spanning hydrophobic α-helices into cell-size-mimicking GUVs. The function of PR in GUVs is also drastically reduced when the N-terminal domain is absent. It seems likely that this drop in function is owing to a reduction in protein insertion and subsequent increase in aggregation. The large volume/surface area ratio of GUVs has previously been shown to affect the spontaneous insertion and function of another α-helical membrane protein, EmrE, when synthesized *de novo* using the PURE system, but the protein-inherent mechanisms underpinning localization remained unexplored (Soga et al., 2014). It should be noted that a recent *in vivo* study identified the N-terminal domain of PR as important for efficient biogenesis even in cells containing chaperoning and insertion pathways (Soto-Rodriguez and Baneyx, 2019). This hints at the intriguing possibility that thermodynamically driven membrane affinity mechanisms aid membrane protein biogenesis *in vivo* to this day, at least for microbial rhodopsins, although this idea has yet to be explored.

Using a well-defined minimal system, we could show that the localization effect mediated by the N-terminal domain of PR does not require additional cellular factors such as SRP, FtsY, or other chaperones and efficiently recruits translating ribosomes to the membrane. It should be noted that the levels of translation were markedly lower for PRΔN. This was likely owing to protein misfolding and aggregation when the N-terminal domain was absent and translating ribosomes were not efficiently attached to the membrane. Because the N-terminal domain alone did not seem to penetrate deeply into the membrane, it is likely that it recruits the ribosomes through interaction with the lipid interface region. We cannot entirely rule out the possibility that the N-terminal domain fully penetrates the bilayer as the resolution of our nile red assay may be limiting.

Our experiments with TM1 suggest that this N-terminal interaction also guides the insertion of the downstream helices of PR, which would otherwise tend to aggregate in the aqueous interior of GUVs. Previous studies have implicated the bilayer interface as an important chemical environment that facilitates the adoption of α-helical structure and subsequent partitioning of hydrophobic peptides into the bilayer interior (Ulmschneider et al., 2011, 2018; White et al., 2001). It is an intriguing possibility that the N-terminal hydrophobic regions of other membrane proteins beyond the rhodopsins also have a high affinity for this region of the bilayer. Such affinity may even act in parallel to SRP-mediated targeting to enhance the efficiency of membrane protein biogenesis. Recent work has shown that SRP in both prokaryotes and eukaryotes recognizes hydrophobic helices along the entire length of the nascent chain and is not specific, as previously thought, for the N-terminal helix (Chartron et al., 2016; Costa et al., 2018; Schibich et al., 2016). Further research is clearly required to address these exciting questions.

Crucially, the N-terminal-dependent localization and insertion of PR can be mimicked by direct, pretranslational binding of ribosomes to the lipid membrane. Given that PR shows moderate functionality in the absence of its N-terminal domain, it seems reasonable to assume that this reduced function is due to reduced protein insertion and increased aggregation as seen in Figure 1. While the translation of PRΔN is markedly lower than full-length PR, there is likely a modest increase in the efficiency of translation following membrane tethering of ribosomes, possibly owing to a reduction in protein aggregation at the exit tunnel of the ribosome, although we cannot be sure of the precise reason for this observation. A recent study has shown that tethering an emerging nascent chain to the membrane increases insertion (Ando et al., 2018). However, these techniques are limited owing to the retention of the N-terminus on the extravesicular surface. Our study builds on this work by attaching ribosomes to the membrane and by using a more realistic, cell-size-mimicking model (GUVs). As mentioned previously, membrane tethering of ribosomes is a well-known strategy used by mitochondria to support effective biogenesis of inner membrane α-helical proteins encoded by the mitochondrial genome (Bonnefoy et al., 2009; Szyrach et al., 2003). Our work presents proof that this strategy can be replicated for synthetic membrane protein assembly in a cell-size-mimicking vesicle system. The technique can now be used to generate simple artificial cell models requiring the insertion of aggregation-prone membrane proteins into vesicle membranes.

In conclusion, we have identified an inherent mechanism of the N-terminal domain of PR that is both necessary and sufficient for the cotranslational recruitment of ribosomes to the membrane of minimal cells. This recruitment then drives the subsequent insertion of downstream α-helices. Our findings using a single model protein hint at a possible explanation for effective membrane protein biogenesis during early evolution in a “pretranslocon” era, and they offer new opportunities for generating artificial cells from the bottom-up. It will be interesting in the future to investigate further examples of this phenomenon and whether such fundamental processes still play a role in membrane protein biogenesis that is masked by the existence of chaperoning systems in modern cells.

### Limitations of the study

Our study used the model membrane PR to delimit requirements for membrane insertion in a simple protocell model. To broaden the impact of this research further testing should be carried out to determine whether and which of the reported observations also apply to other membrane proteins and how they are integrated with more complex integration machineries in living cells. Furthermore, in GUVs, we were not able to quantify the kinetics or the efficiency of the insertion process. Kinetics of membrane protein folding in a minimal context has been previously investigated (Harris et al., 2017) and was beyond the scope of our study. Determining insertion efficiencies within GUVs was not possible owing to the lack of robust methodologies for achieving a relevant result. It is likely that the simple protein-inherent process presented here has much lower insertion efficiency than translocon-mediated mechanisms.

### Resource availability

#### Lead contact

Further information and requests should be directed to and will be fulfilled by the Lead Contact, Ross Eaglesfield (ross.eaglesfield@glasgow.ac.uk).

#### Materials availability

All plasmids used in this study are available upon reasonable requests from the lead contact. Requests relating to *E. coli* strain JE28 should be directed to S.S.

#### Data and code availability

All raw images and data generated and analyzed during this study are available from the lead contact upon request.

## Methods

All methods can be found in the accompanying transparent methods supplemental file.

## Supplemental information

Document S1. Transparent methods and Figures S1–S11
