# Supplementary material for: Cotranslational recruitment of ribosomes in protocells recreates a translocon-independent mechanism of proteorhodopsin biogenesis
Source: iScience. 2021 Apr 20;24(5):102429. doi: 10.1016/j.isci.2021.102429 (PMC8102411; doi:10.1016/j.isci.2021.102429)
Supplement: Document S1. Transparent methods and Figures S1–S11 [file mmc1.pdf]

iScience, Volume *24*

Supplemental information

Cotranslational recruitment of ribosomes in protocells recreates a translocon-independent mechanism of proteorhodopsin biogenesis

Ross Eaglesfield, Mary Ann Madsen, Suparna Sanyal, Julien Reboud, and Anna Amtmann

Supplemental Information

Supplemental Figures

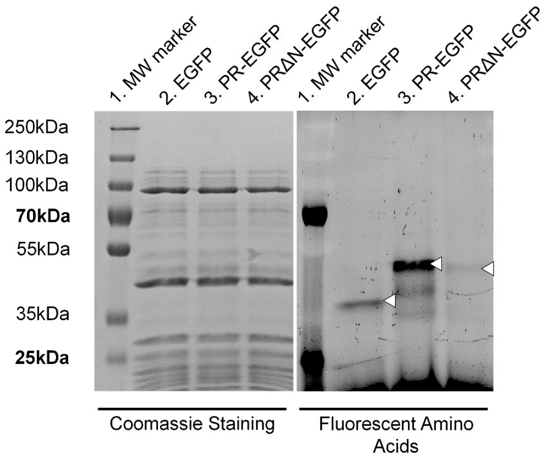

Figure S1. SDS-PAGE analysis of batch PURE reactions. Related to Figure 1. Proteins EGFP, PR-EGFP and PRΔN-EGFP were synthesized in bulk in the presence of large unilamellar vesicles (LUVs) and BODIPY-labelled lysine amino acids. Following protein synthesis reactions were run on an SDS-PAGE gel and imaged using the fluorescence of incorporated BODIPY-labelled lysine and coomassie staining. Translation products are marked with white arrows.

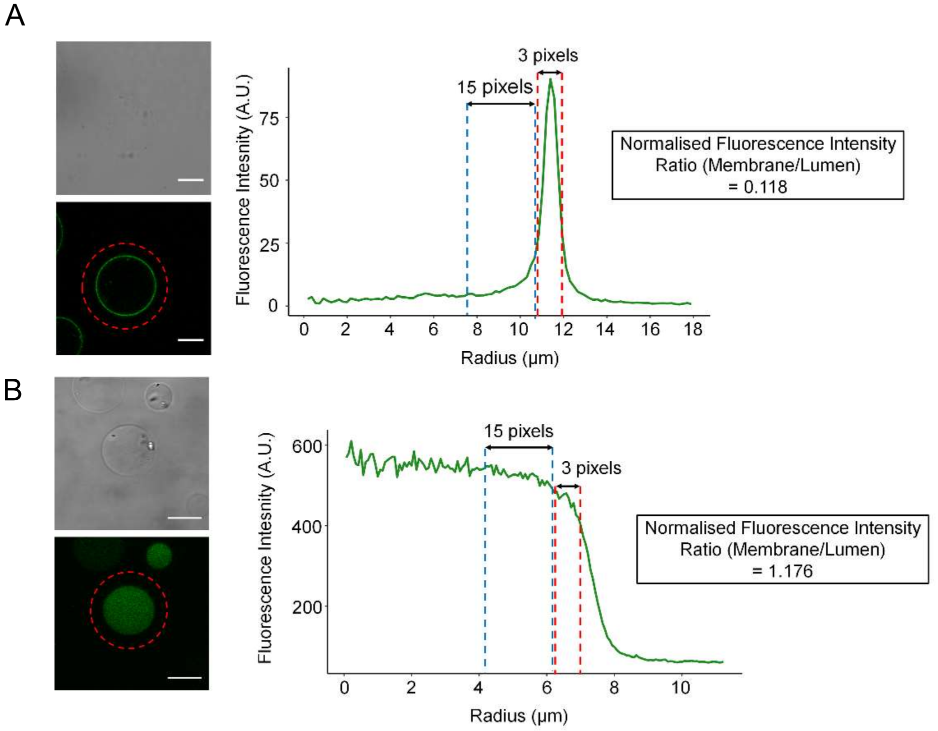

Figure S2. Examples of radial profile fluorescence analysis. Related to Figure 1. (A) Confocal microscopy image of a GUV with membrane localized fluorescence. The red circle indicates the region of interest used for radial profiling. Scale bar is 10 μm. The corresponding line plot shows the radial fluorescence profile for the image shown. Membrane fluorescence was determined by calculating an average of three pixels with the highest signal being the middle pixel. Lumen fluorescence was determined by taking an average of the subsequent 15 pixels moving into the vesicle lumen. Values were normalized to background fluorescence measured as an average of three arbitrary points on the image without GUVs present. **(B)** Confocal microscopy image of a GUV with lumen localized fluorescence. The red circle indicates the region of interest used for radial profiling. Scale bar is 10 μm. The corresponding line plot shows the radial fluorescence profile for the image shown. When no membrane fluorescence peak was observed the average of three pixels was taken at the point where fluorescence stopped increasing initially. Lumenal fluorescence was determined as before by averaging the fluorescence intensity of 15 pixels further into the vesicle lumen. Background adjustments were carried out as before. All images were captured at a resolution of 2048 x 2048 in order to maintain a consistent pixel size.

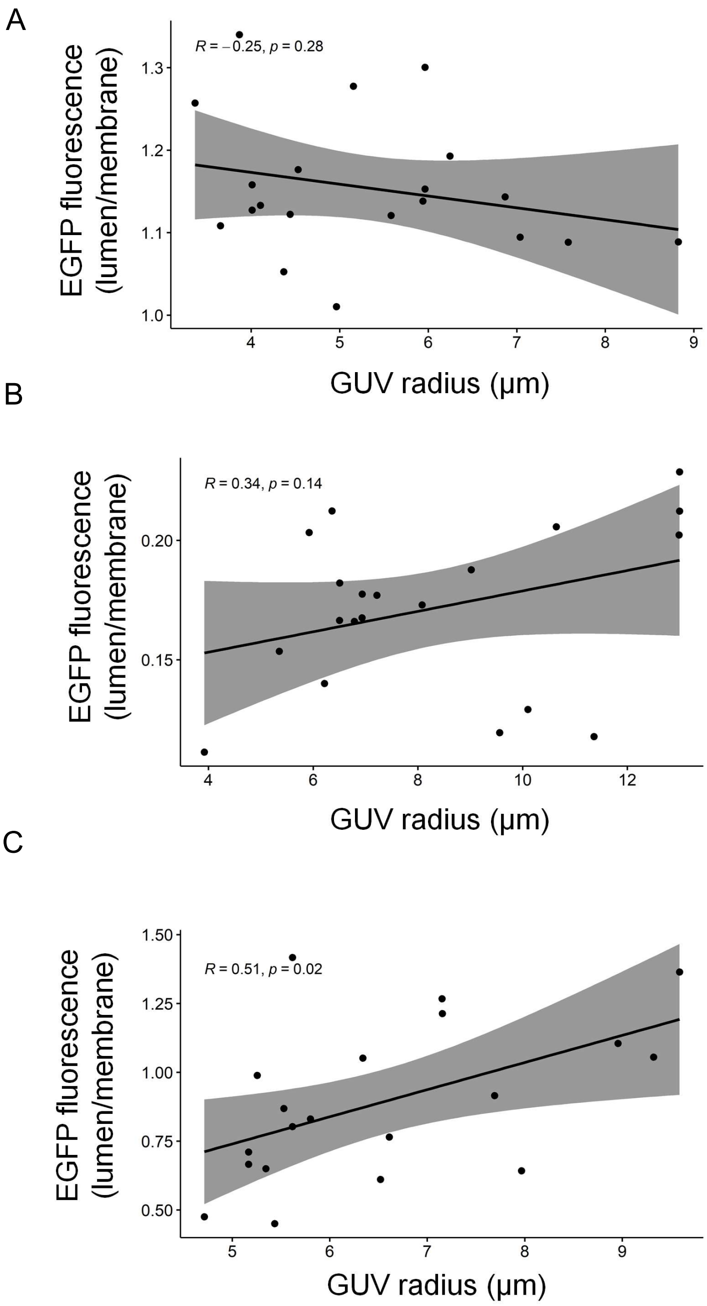

Figure S3. Correlation analysis of membrane localized EGFP and GUV size. Related to Figure 1. Fluorescence ratios from Fig. 1E were plotted against GUV radius and a pearson correlation analysis was performed. The resulting model is shown with a 95% confidence interval. **(A)** EGFP **(B)** PR-EGFP **(C)** PRΔN-EGFP.

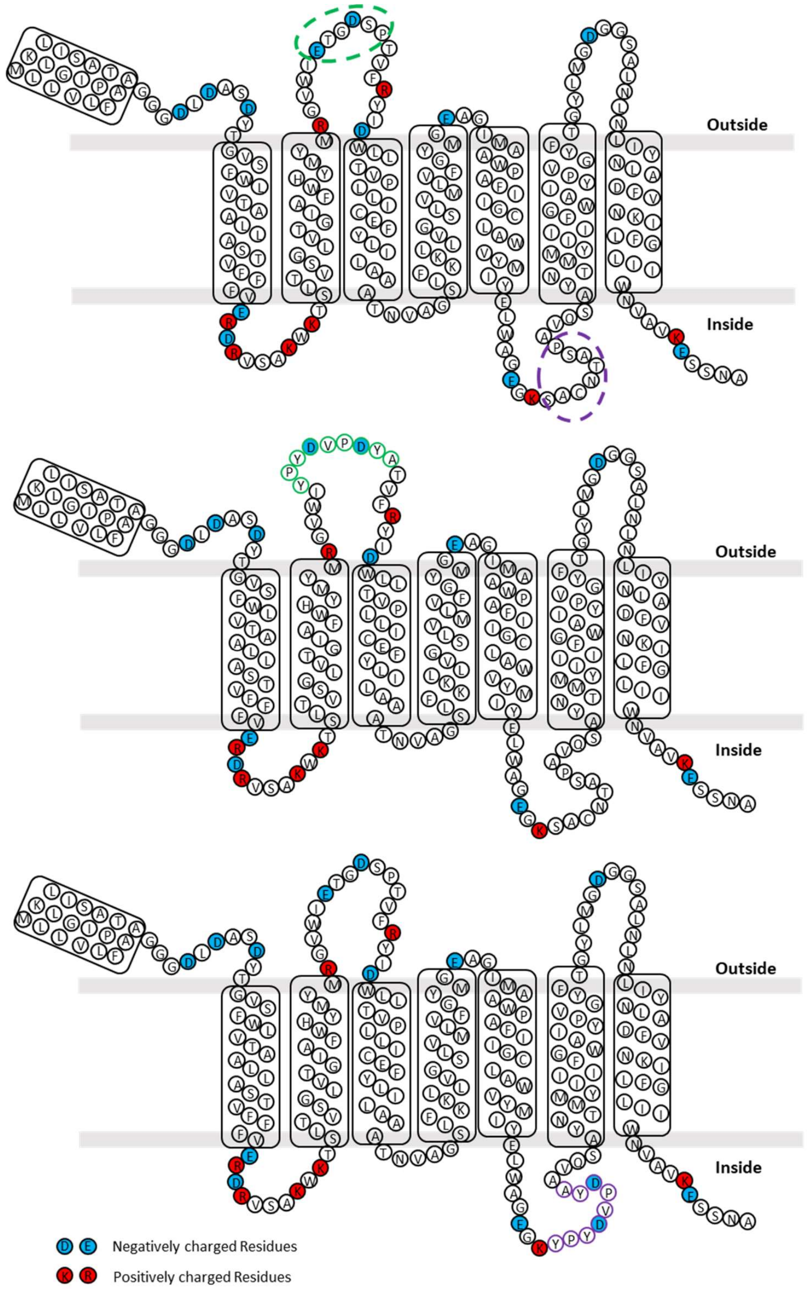

Figure S4. Extra- and intracellular loop modification of PR with hemagglutinin epitope. Related to Figure 1. The topological structure of PR is shown with the N-terminal hydrophobic domain shown on the left followed by the 7 transmembrane helices. HA epitope insertion by Gibson assembly yielded the extracellular (green) modification in PR-EL1HA and the intracellular (purple) modification in PR-IL3HA. Negatively charged residues are blue and positively charged residues are red.

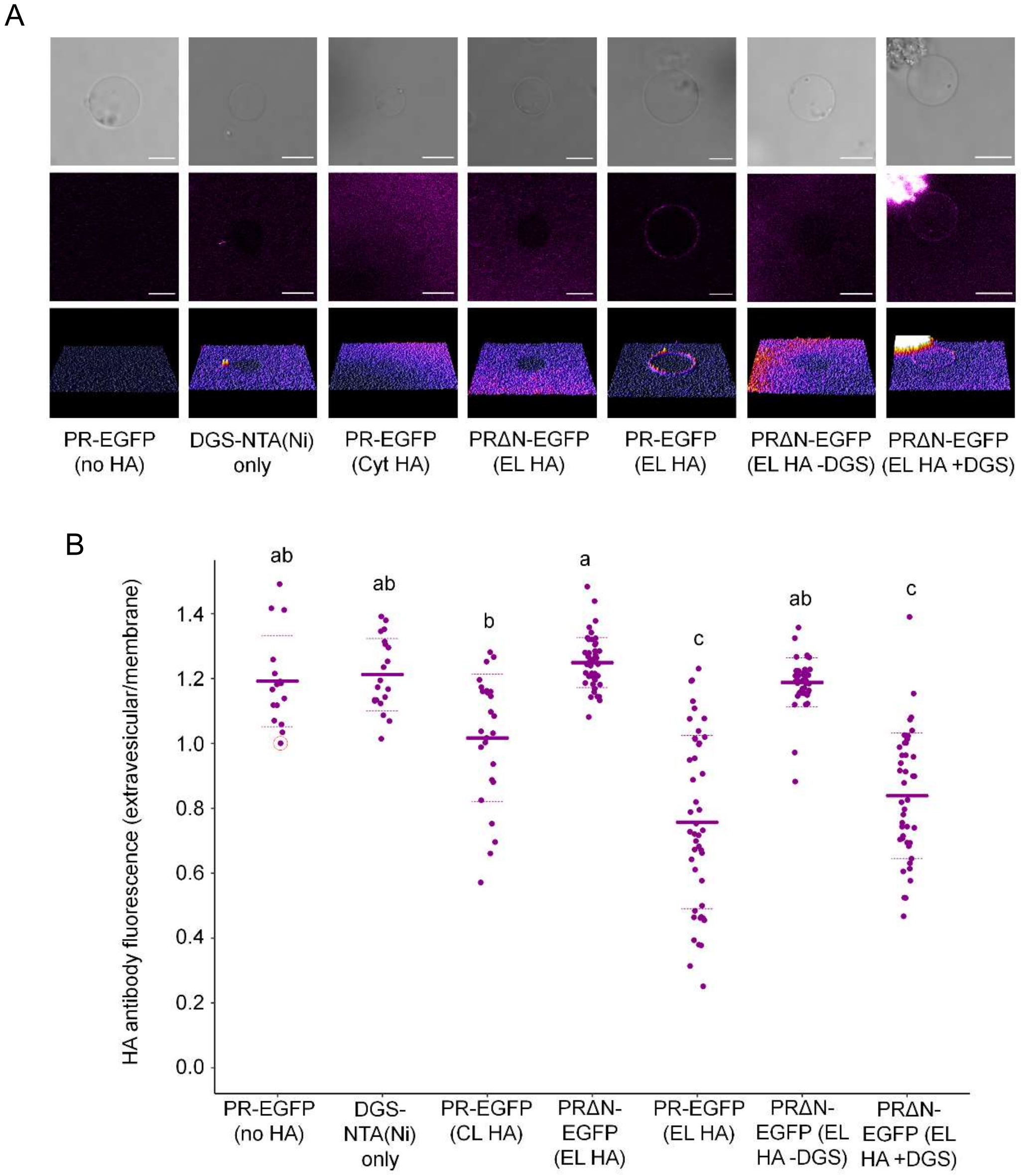

Figure S5. Data for Alexa Fluor-647 conjugated HA antibody-binding assays. Related to Figure 1. (A) Confocal images of GUVs for each protein construct and condition. Images shown are brightfield, fluorescence emission > 650nm and 3-dimensional representations of fluorescence. Scale bars are 10 µm. DGS-NTA(Ni) only sample does not contain a PURE reaction but was used as a control to show that antibody could not bind directly to the NTA containing lipid. **(B)** Extravesicular/membrane fluorescence intensity ratio derived from radial profiles of Alexa Fluor-647 conjugated HA antibody fluorescence of individual GUVs (n = 16 – 48). Mean values (solid lines) and standard deviations (dashed lines) are based on individual GUVs (filled circles). Different letters represent statistically significant differences ($p < 0.001$; one-way ANOVA using Tukey (HSD) post-hoc analysis). The lowest ratio detected in control vesicles lacking an HA epitope is marked in red and was used to normalize all subsequent data.

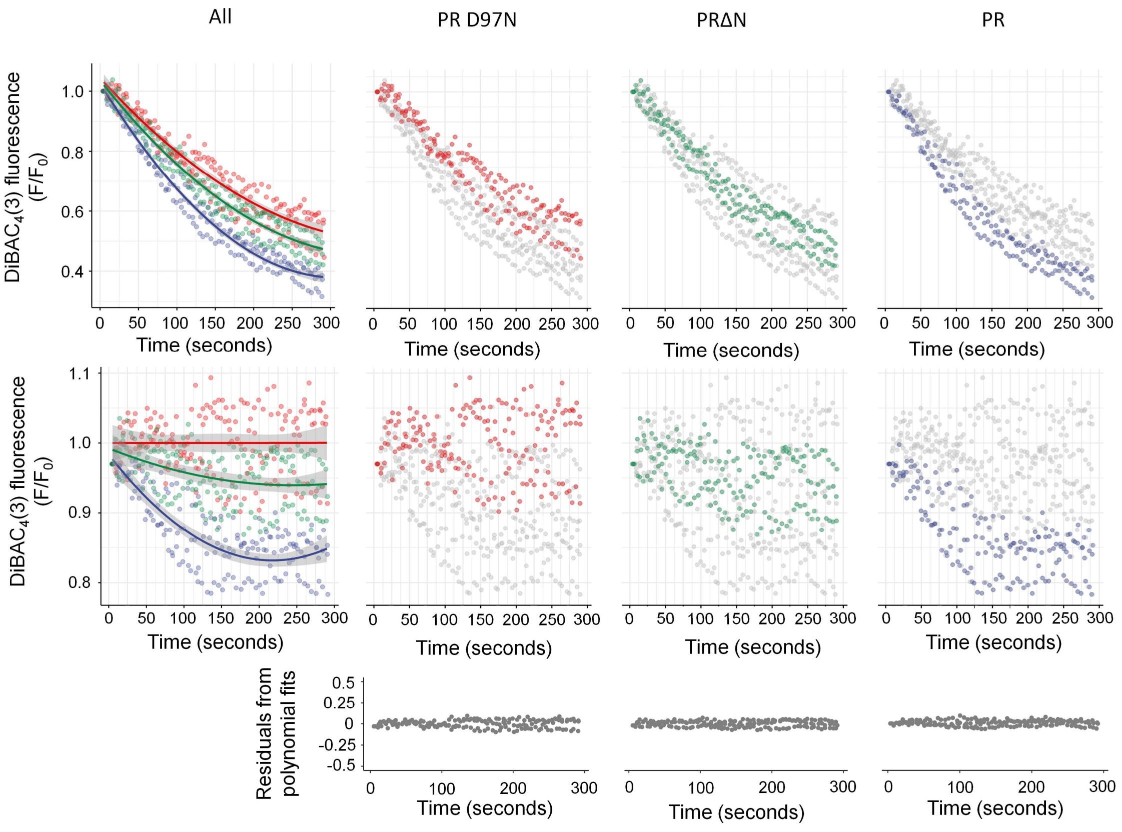

Polynomial used for normalisation: $y = 3.654 \times 10^{-6}x^2 - 2.823 \times 10^{-3}x + 1.044$

$R^2 = 0.616$

Figure S6. Functionality of PR synthesized inside GUVs. **Related to Figure 1.** Fluorescence intensity of $DiBAC_4(3)$ in the membrane of GUVs containing a non-functional PR mutant (D97N), PR without the N-terminal domain (PRΔN) and full-length PR. Individual vesicles were analyzed every 5 seconds under constant excitation with a 488nm laser to excite both $DiBAC_4(3)$ and PR. 12 GUVs from 3 individually prepared experiments were analyzed for each protein construct with results shown as the mean value for each experiment. Panel 1 shows all data points with fitted 2nd order polynomials and 99% confidence intervals. Red represents PR D97N, green PRΔN and blue PR. The remaining panels show individual data points for all protein constructs with individual protein constructs highlighted according to color as in panel 1. The top row shows raw data with the second row showing data normalised to the displayed polynomial function for PR D97N to account for photobleaching of $DiBAC_4(3)$. Residuals for each fitted polynomial function are shown under the respective panel.

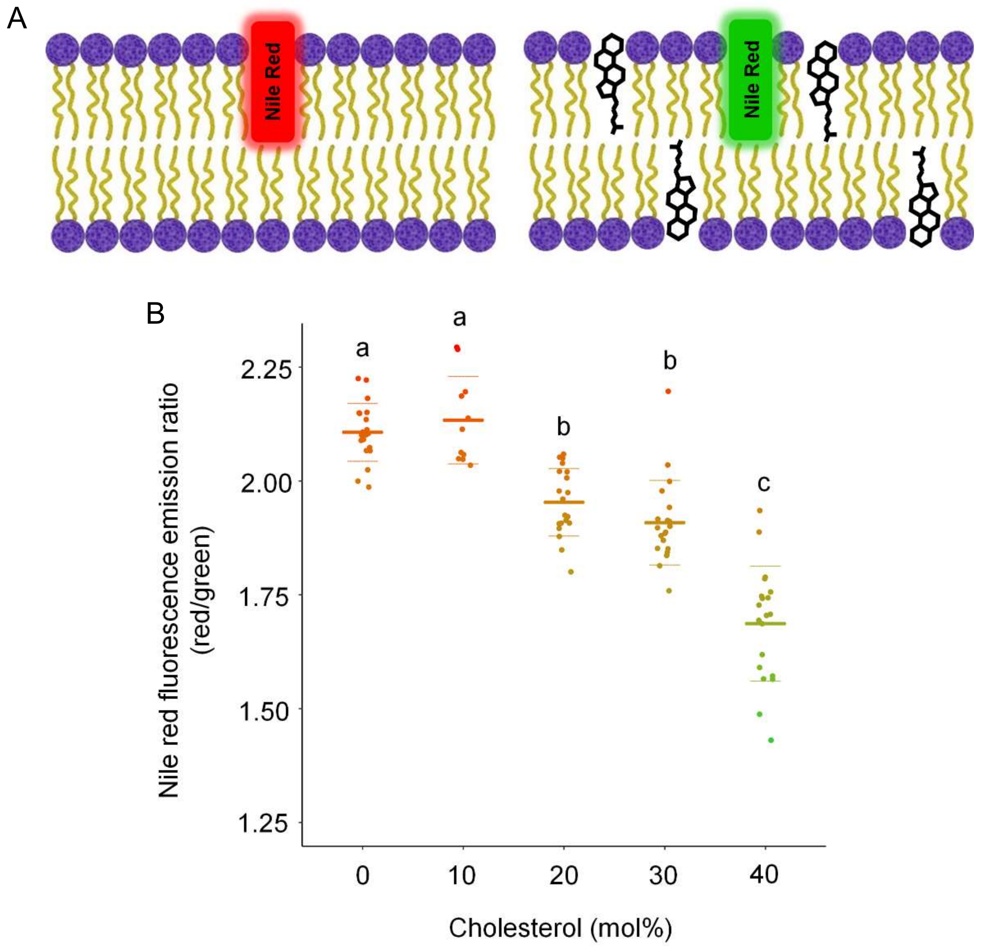

Figure S7. Nile red calibration in POPC:cholesterol GUVs. Related to Figure 2. (A) Schematic showing the nile red fluorescence emission response to increased cholesterol and thus lipid order. Loosely packed lipids without cholesterol lead to probe emission being red-shifted. Increasing concentrations of cholesterol leads to a blue-shift in the emission spectrum due to increased lipid tail order. **(B)** Quantification of nile red fluorescence ratio (red/green) for GUVs containing increasing concentrations of cholesterol. Mean values (solid lines) and standard deviations (dashed lines) are shown for ≥11 individual GUVs (filled circles). Different letters represent statistically significant differences ($p < 0.001$; one-way ANOVA using Tukey (HSD) post-hoc analysis).

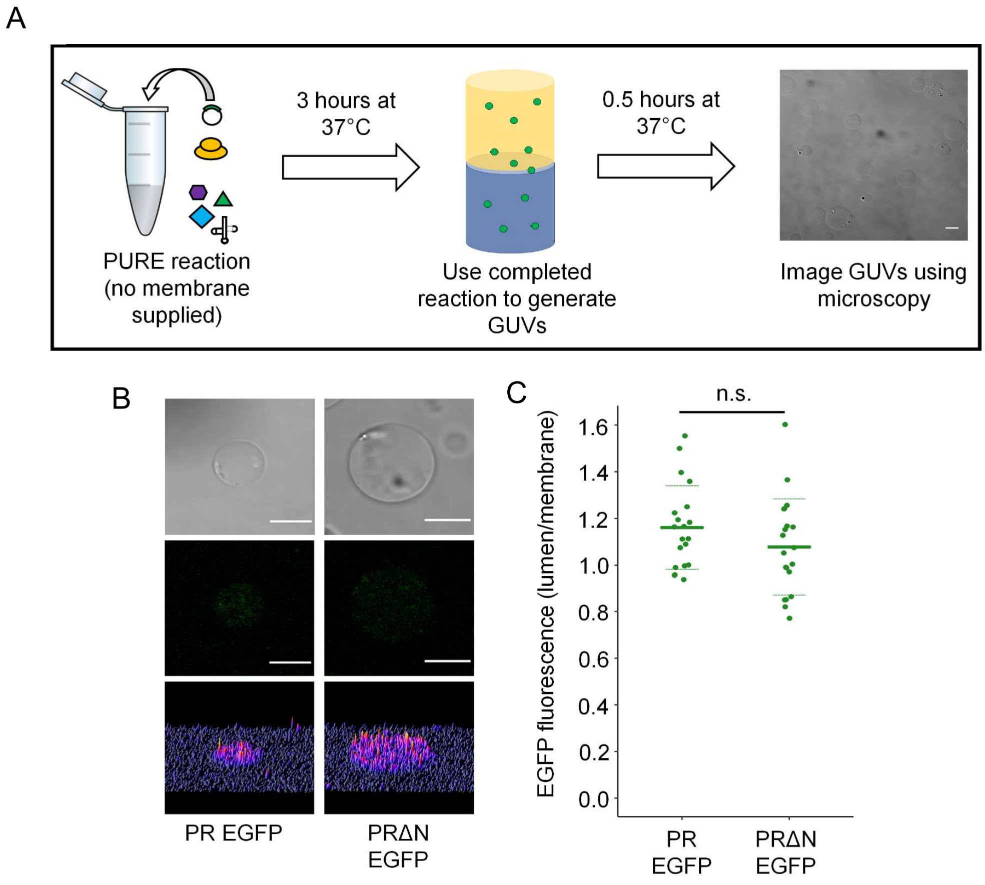

Figure S8. Membrane recruitment and insertion of PR does not occur post-translationally. Related to Figure 3. (A) Schematic representation of the experimental design used to investigate post-translational protein localization. Bulk reactions were performed without lipids. Following completion of reactions solutions were used as the inner solution to generate GUVs by droplet transfer. Following a short incubation, GUVs were visualized using confocal microscopy. **(B)** Confocal image showing GUVs encapsulating PR-EGFP and PRΔSP synthesized prior to vesicle formation without an amphiphile present. Scale bars are 10 μm. **(C)** Lumen/membrane fluorescence intensity ratio derived from radial profiles of EGFP fluorescence emission of individual GUVs. Mean values (solid lines) and standard deviations (dashed lines) are shown for 20 individual GUVs (filled circles). n.s. represents no statistical significance (P = 0.182) using a two-sample t-test assuming equal variance.

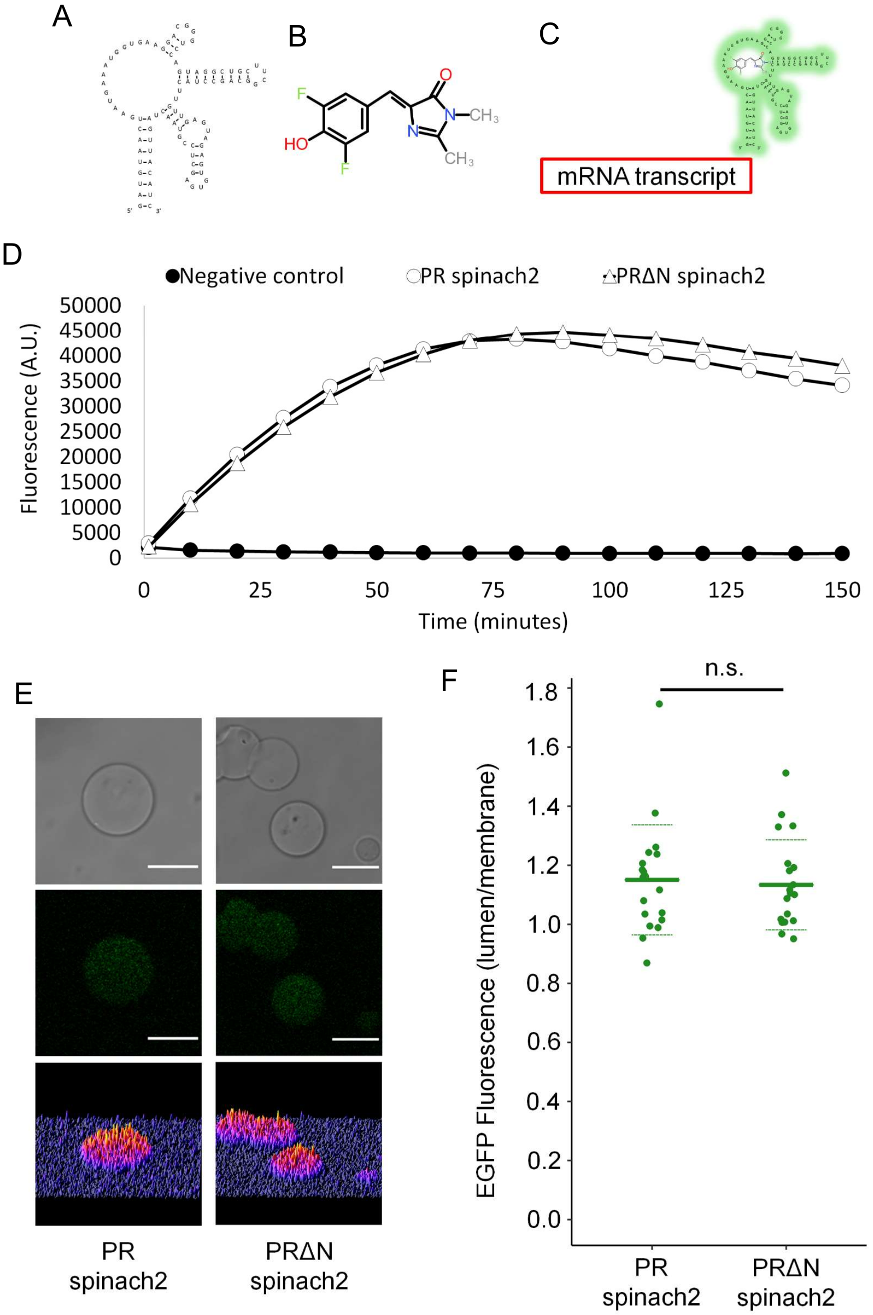

Figure S9. Spinach2-tagged mRNA is localized diffusely throughout the lumen of GUVs following transcription. Related to Figure 3. (A) Sequence and secondary structure of the RNA aptamer spinach2. **(B)** Chemical structure of the conditionally fluorescent dye 3,5-difluoro-4-hydroxybenzylidene imidazolinone (DHFBI). **(C)** Spinach2 aptamer attached to the 3' end of mRNA transcripts. **(D)** Fluorescence time-series showing fluorescence of PR-spinach2 (open circles), PRΔN-spinach2 (open circles) and a negative control containing no template DNA (filled circles). **(E)** Confocal image showing GUVs after mRNA synthesis and treatment with DHFBI. Scale bars are 10 µm. and **(F)** Lumen/membrane fluorescence intensity ratio derived from radial profiles of DHFBI fluorescence emission of individual GUVs. Mean values (solid lines) and standard deviations (dashed lines) are shown for 20 individual GUVs (filled circles). n.s. represents no statistical significance (P = 0.756) using a two-sample t-test assuming equal variance.

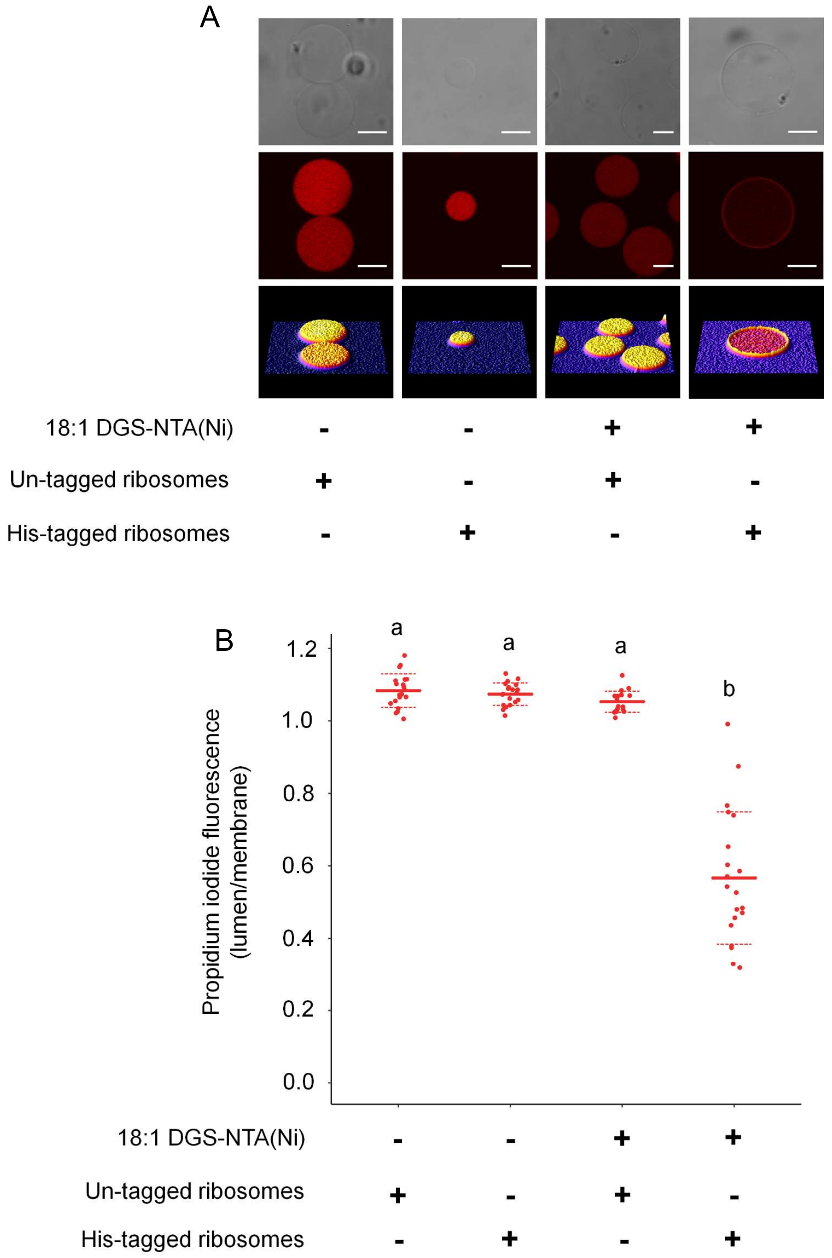

Figure S10. Synthetic attachment of histidine-tagged 70S ribosome complexes to GUV membranes using nitrilotriacetic acid (NTA) affinity. Related to Figure 4. (A) Confocal microscopy images of GUVs encapsulating 1 μM of 70S ribosomes stained with 100 μg/ml propidium iodide (PI). GUVs were generated with and without 2.7 mol% DGS-NTA(Ni) in the membrane. Scale bars are 10 μm. **(B)** Lumen/membrane ribosomal fluorescence intensity ratio derived from radial profiles of PI emission in individual GUVs. Mean values (solid lines) and standard deviations (dashed lines) are based on 30 individual GUVs (filled circles). Different letters represent statistically significant differences ($p < 0.001$; one-way ANOVA using Tukey (HSD) post-hoc analysis).

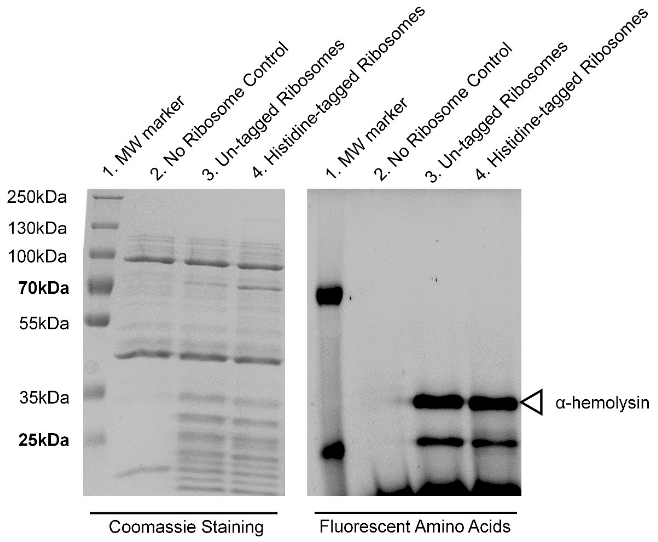

Figure S11. Purified, histidine tagged ribosomes are functional in the PURE system. Related to Figure 4. SDS-PAGE analysis of PURE reactions containing no ribosomes (lane 2), 1 μM kit supplied ribosomes (lane 3) or 1 μM self-purified, histidine-tagged ribosomes (lane 4). Reactions were supplemented with pDNA encoding α-hemolysin and fluorescently-tagged lysine amino acids. The resulting gel was probed using coomassie (left hand image) and BODIPY fluorescence (right hand image).

Transparent Methods

Chemicals

All lipid products were purchased from Avanti Polar Lipids. Alexa Fluor-conjugated antibodies were purchased from Thermo Fisher Scientific. All other chemicals were purchased from Sigma-Aldrich unless otherwise stated.

Cloning procedures

Plasmid DNA containing the gene encoding green-light absorbing proteorhodopsin from the SAR86 group of marine γ-proteobacteria was kindly provided by Prof. Edward DeLong (Beja et al., 2000). The vector backbone used for the construction of all cell-free expression constructs was the control plasmid supplied with the PURExpress kit (New England Biolabs). This vector contains a T7 promoter, ribosome binding site and T7 terminator sequence with an ampicillin resistance gene for selection. The PR encoding gene was amplified by PCR to remove the endogenous stop codon and was ligated into the cell-free expression vector between KpnI and BamHI (for fusion constructs) or XhoI (for PR only constructs) restriction sites. Meanwhile, PR truncations were introduced by PCR with a start codon replacing each removed residue. These sequences were then ligated into the expression vector using the same restriction sites as the full-length construct. The gene encoding EGFP was then amplified by PCR and inserted downstream of both full-length PR and PR truncations in between BamHI and XhoI restriction sites. For EGFP only plasmids, PR was removed by PCR before re-ligation of the linearised vector. The 84-residue encoding linker sequence of tolA was amplified by PCR directly from E. coli colonies and was ligated downstream of PR and PRΔN between BamHI and SphI restriction sites. The 17-residue encoding sequence of secM was also amplified directly from E. coli and ligated downstream of tolA between SphI and XhoI restriction sites.

The sequence for the mRNA aptamer Spinach2 was 5' – GATGTAACTGAATGAAATGGTGAAGGAC GGGTCCAGTAGGCTGCTTCGGCAGCCTACTTGTTGAGTGAGTGTGAGCTCCGTAACTAGTTACA TC- 3' and was amplified directly from DNA oligonucleotides (Integrated DNA Technologies) by PCR and inserted at the 3' end of PR and PRΔN constructs between BamHI and XhoI restriction sites.

The sequence encoding the HA epitope was 5' -TATCCGTATGATGTGCCGGATTATGCG- 3' and was used directly from DNA oligonucleotides (Integrated DNA Technologies) with 20 base pair complementary overhangs for Gibson Assembly reactions to generate PR and PRΔN constructs with HA epitopes in the second periplasmic/extracellular loop and the third intracellular loop of PR. All constructs were confirmed by sequencing prior to experimental use.

Bulk cell-free protein synthesis using the PURE system

Cell-free reactions were carried out using the PUREfrex1.0 (GeneFrontier) cell-free transcription-translation system. Reaction volumes used for bulk synthesis were 10 µl and were set up according to the manufacturer's protocol. Each reaction was supplemented with 0.3 µl of FluoroTect™ GreenLys in vitro translation labelling system (Promega), 0.5 µl of Murine RNase inhibitor (New England Biolabs), 20 ng µl-1 of plasmid DNA and 1 mg/ml of POPC (Avanti Polar Lipids) LUVs. LUV stocks (10 mg/ml in 50 mM HEPES-KOH (pH 7.6), 100 mM potassium glutamate and 15 mM magnesium acetate) were generated by manual extrusion through 0.1 µm polycarbonate membranes using a mini-extruder (Avanti Polar Lipids). Reactions for PR constructs were supplemented with 100µM all-trans-retinal. Reactions were incubated at 37°C for three hours in the dark. SDS-PAGE loading buffer (4x) was then added directly to reaction mixtures and samples were incubated for 15 minutes at 65°C to avoid the formation of precipitates. Samples were then used directly for SDS-PAGE. In-gel fluorescence from labelled amino acids was analysed using a Typhoon FLA 9000 (GE Healthcare) with an excitation wavelength of 473 nm and a long-pass emission filter for detection of wavelengths > 510 nm. Gels were then recovered and stained with coomassie to confirm the presence of the protein components of the PURE system.

Preparation of giant unilamellar vesicles (GUVs) and cell-free reaction encapsulation

GUVs were generated using the droplet transfer method as previously described (Altamura et al., 2017; Noireaux and Libchaber, 2004). An aqueous/lipid interface was generated by gently layering 300 µl of 0.5 mM POPC (Avanti Polar Lipids) solubilized in light mineral oil on top of an outer aqueous solution supplemented with 200 mM glucose (see below for solution make-up). The resulting two-phase solution was left at room temperature for 2 hours to allow saturation of the interface with POPC molecules. Meanwhile, 20 µl of an inner solution supplemented with 200 mM sucrose was prepared and transferred into 600 µl of fresh 0.5 mM POPC in light mineral oil. This solution was then emulsified by pipetting for 30 seconds and gently layered on top of the previously prepared, now lipid saturated, aqueous/lipid interface. The entire mixture was then centrifuged at 2,500 x g for 10 minutes and pelleted GUVs were collected using a pipette following the careful removal of the oil phase.

For cell-free reactions, the inner solution was composed of the PURE cell-free reaction mixture supplemented with 200 mM sucrose, 1 µl murine RNase inhibitor (New England Biolabs) and 20 ng µl-1 of the relevant plasmid DNA. Additionally, for cell-free reactions with His-tagged ribosomes, the supplied ribosome solution was omitted and 1 µM of self-purified His-tagged ribosomes was added (see below for purification and labelling methods). The outer solution was composed of the small molecular weight components of the PURE system (0.3 mM of each of the 20 amino acids, 1.5 mM spermidine, 3.75 mM ATP, 2.5 mM GTP, 1.25 mM of both CTP and UTP, 25 mM creatine phosphate, 1.5 mM 1,4-dithiothreitol (DTT), 18 mM magnesium acetate, 280 mM potassium glutamate, 50 mM HEPES-KOH pH7.6 and 0.02 mg ml-1 folinic acid), supplemented with 200 mM glucose and osmotically matched to the inner solution with NaCl (typically 75 mM) using a Vapro 5520 vapor pressure osmometer (Wescor). Following collection, GUVs were incubated for three hours at 37°C in the dark. All reactions involving the synthesis of PR or PRΔN were supplemented with 100µM all-trans-retinal following GUV collection and prior to incubation.

For ribosome binding experiments the inner solution was composed of 100 mM potassium glutamate, 18 mM magnesium acetate, 50 mM HEPES-KOH pH7.6, 1 µM 70S ribosomes and 200 mM sucrose. The outer solution was composed of 100 mM potassium glutamate, 18 mM magnesium acetate, 50 mM HEPES-KOH pH7.6 and 200 mM glucose. GUVs were collected and visualised immediately.

For mRNA localisation experiments, ribosomes were omitted from the reaction mixture to avoid translation of mRNAs and 3,5-difluoro-4-hydroxybenzylidene imidazolinone (DHFBI) was added to a final concentration of 20 µM in the inner and outer aqueous solutions. Following collection GUVs were incubated at 37°C for one hour before visualization.

DiBAC4(3) fluorescence quenching assay

PURE encapsulating GUVs were prepared as previously described and incubated for 20 minutes in the dark at 37°C. GUV suspensions were then transferred to 4°C and were either used immediately or stored for no more than 2 hours. DiBAC4(3) was added to a final concentration of 1 µM and vesicle suspensions were allowed to settle in microscopy chambers for 20 minutes in the dark prior to imaging. GUVs containing PR D97N, PRΔN and PR were then excited with a 488 nm argon laser for excitation of both DiBAC4(3) and PR. Vesicles were constantly excited for 290 seconds with quantification of membrane bound DiBAC4(3) fluorescence performed every 5 seconds.

Hemagglutinin (HA) antibody binding assay

GUVs collected following synthesis of the relevant protein were re-suspended in fresh outer solution supplemented with 1 mg ml-1 of Alexa Fluor 647-conjugated HA antibody (Thermo-Fisher Scientific). The solution was mixed gently by pipetting and was incubated at 37°C for 30 minutes. GUVs were then pelleted by centrifugation at 2,500 x g for 10 minutes at room temperature, antibody-containing solution was carefully removed, and GUVs were re-suspended in fresh outer solution.

Affinity purification of histidine-tagged ribosomes and fluorescent labelling of ribosomal proteins

E. coli strain JE28 was used for isolation of tetra-histidine-tagged 70S ribosomes using a previously established protocol (Ederth et al., 2009). Briefly, JE28 cultures were grown shaking at 37°C in LB media supplemented with 50 µg ml-1 kanamycin. At OD600 = 1 cultures were slowly cooled to 4°C. Cells were then pelleted by centrifugation at 4,000 rpm for 30 minutes at 4°C and re-suspended in buffer A (20 mM HEPES-KOH pH 7.6, 10 mM magnesium chloride, 150 mM potassium chloride and 30 mM ammonium chloride) supplemented with 0.5 mg ml-1 lysozyme and 10 µg ml-1 RNase-free DNase I (Promega). Cell suspensions were further lysed by sonication. Cell lysates were subjected to two rounds of centrifugation at 20,000 x g for 30 minutes at 4°C to remove insoluble material. Cleared lysates were then applied to a pre-packed Ni-NTA column equilibrated with un-supplemented buffer A. The column was washed with buffer A containing 5 mM imidazole before ribosomes were eluted with buffer A containing 150 mM imidazole. Pooled elution fractions were dialyzed twice for 2 hours against 5 L of buffer A at 4°C to remove imidazole. At this stage ribosomes were either pelleted by ultracentrifugation through a 30 % (w/v) sucrose cushion at 130,000 x g for 16 hours at 4°C and re-suspended in polymix buffer (5 mM magnesium acetate, 5 mM ammonium chloride, 95 mM potassium chloride, 0.5 mM calcium chloride, 8 mM putrescine, 1 mM spermidine, 5 mM potassium phosphate (pH 7.6) and 1 mM DTT), or were fluorescently labelled (see below). Ribosome concentration was determined by measuring absorbance at 260 nm and using an extinction coefficient of 3.91 × 107 M-1 cm-1 (Becker et al., 2012). Ribosomes were further concentrated to 20 µM using 10,000 Da MWCO concentration columns (BioVision).

For fluorescent labelling, Alexa Fluor 488 5-SDP ester (Thermo-Fisher Scientific) was added to 5 ml of imidazole-free ribosome solution at a concentration of 50 µg ml-1. The resulting solution was gently mixed at room temperature for one hour and then dialyzed against 5 L of dye-free solution for 2 hours at 4°C. This solution was then ultracentrifuged through a 30 % (w/v) sucrose cushion at 130,000 x g for 16 hours at 4°C. The resulting pellet containing fluorescently labelled ribosomes was re-suspended in polymix buffer and concentrated to 20 µM as before.

Nile red lipid order assay

GUVs collected following synthesis of the relevant protein using the PURE system were re-suspended in fresh PURE outer solution supplemented with 200 mM glucose and 0.1 µM nile red before being visualised using confocal microscopy. A 488 nm argon laser was used to excite nile red and emission was collected using band-pass filters for 510 - 590 nm and for 650 - 750 nm.

Microscopy

Confocal microscopy images were taken using a Leica TCS SP8 laser scanning confocal microscope equipped with a 63x oil immersion objective. Samples were placed in 8-well, uncoated polymer µ-slides (Ibidi) and allowed to settle to the bottom of the chambers for 10 minutes prior to imaging. Image analysis was performed using Fiji software equipped with a plugin to measure radial fluorescence profiles. Three-dimensional surface plots using a fire LUT were also generated using Fiji in order to enhance the visualization of samples with low fluorescence. Background adjustments were performed by taking three background readings from arbitrary points on each image and calculating the mean intensity.

Statistical analysis

Tests performed for statistical analysis were one-way ANOVA with Tukey (HSD) post-hoc analysis and two-tailed, two sample Student’s t-test. All statistical tests were performed using R version 3.4.3 and statistical significance was assumed with a p-value of < 0.001 unless otherwise stated.

Supplemental References

Altamura, E., Milano, F., Tangorra, R.R., Trotta, M., Omar, O.H., Stano, P., and Mavelli, F. (2017). Highly oriented photosynthetic reaction centers generate a proton gradient in synthetic protocells. Proceedings of the National Academy of Sciences of the United States of America *114*, 3837-3842.

Becker, M., Gzyl, K.E., Altamirano, A.M., Vuong, A., Urbahn, K., and Wieden, H.-J. (2012). The 70S ribosome modulates the ATPase activity of Escherichia coli YchF. RNA Biology *9*, 1288-1301.

Ederth, J., Mandava, C.S., Dasgupta, S., and Sanyal, S. (2009). A single-step method for purification of active His-tagged ribosomes from a genetically engineered Escherichia coli. Nucleic Acids Research *37*, e15.

Noireaux, V., and Libchaber, A. (2004). A vesicle bioreactor as a step toward an artificial cell assembly. Proceedings of the National Academy of Sciences of the United States of America *101*, 17669-17674.
